# Status and influence of parental rearing style and social psychology process on social adaptability of medical students: using college students in Jilin Province, China, as an example

**DOI:** 10.3389/fpsyg.2025.1617863

**Published:** 2025-10-14

**Authors:** Juan Du, Hongjuan Ge, Wei Nie, Xinrui Feng, Bing Shao

**Affiliations:** ^1^School of Pharmacy, Jilin Medical University, Jilin, China; ^2^School of Public Health, Jilin Medical University, Jilin, China; ^3^Department of Clinical Nutrition, Shengjing Hospital of China Medical University, Shenyang, China

**Keywords:** parental rearing styles, social adaptability, social avoidance, social distress, medical college students

## Abstract

**Background:**

Although social adaptability is crucial for medical students, the combined influence of parental rearing styles and social psychology process on this competency is not well-explored, particularly in the context of China. This study examines their status and relationships among Chinese medical students.

**Methods:**

An online questionnaire survey based on the web-based survey platform “Questionnaire Star” was performed with medical college students in Jilin Province, China. The questionnaire content comprised the status of the participants' parental rearing style, social avoidance and distress, and social adaptability by employing specific survey scales, and mediation effect analysis was conducted to examine the mediating role of social psychology process in the relationship between parental rearing styles and social adaptability.

**Results:**

Two thousand hundred and sixty-six medical college students were subjected to statistical analysis. Parental rearing styles and social avoidance and distress show differences among sociodemographic factors of gender, household registration, whether is one-child, and parental education (*p* < 0.05). Students from urban households, with one-child identities and those whose parents had high educational levels obtained high social adaptability scores (*p* < 0.05). The multivariate analysis results are as follows: social distress (β = −0.399, *p* < 0.001), social avoidance (β = −0.304, *p* < 0.001), mother's rearing style of “emotional warmth and understanding” (β = 0.135, *p* < 0.001), and father's rearing style of “overprotection” (β = −0.087, *p* < 0.001) are the independent factors influencing medical college students' social adaptability. Mediation effect analysis further reveal that parental rearing styles of “emotional warmth and understanding” and “overprotective” can directly or indirectly affect students' social avoidance and distress to influence their social adaptability.

**Conclusion:**

Reducing students' social avoidance and distress, strengthening “emotional warmth and understanding,” and preventing “overprotective” parental rearing styles are effective ways to improve medical college students' social adaptability.

## Background

The responsibility of medical education is to impart medical scientific knowledge or skills to students; however, this is no longer considered sufficient; other qualities are also required. In addition to knowledge and skills, students are now also assessed in terms of performance in practice. Currently, medical education emphasizes promoting students' comprehensive ability and quality (Wijnen-Meijer et al., 2020). Social adaptability refers to individuals' adaptive ability to make psychological, physiological, and behavioral changes to effectively survive and achieve a harmonious state with society; this capability reflects an individual's comprehensive quality (Liu et al., 2023). It represents a critical competency that medical college students are expected to develop, particularly after they graduate from school and transition into their roles as healthcare workers. With the development of society, increasingly fierce social competition has introduced increasingly high requirements for college students' social adaptability, which is the most critical aspect of their entry into society (Chen and Sto, 2021; Ji and Zheng, 2021).

China has the world's largest medical education system. The number of medical graduates has also increased dramatically in recent years (Wang, 2021). Given this background, more medical college students must encounter severe difficulties in seeking employment opportunities after graduation. They must immediately change their identity and adapt to their role as healthcare workers. Performance in terms of social adaptability may determine whether medical students can meet their job requirements and achieve breakthroughs in their future careers.

Traditional medical curricula in China primarily focus on technical knowledge, frequently overlooking the comprehensive training in social adaptability. Chinese medical students typically spend most of their time learning medical knowledge or skills in school, and special opportunities for cultivating and improving their social adaptability are rarely provided for them. Parental rearing style is a collection of stable attitudes exhibited by parents through specific, goal-directed behaviors (e.g., parenting practices) and non-goal-directed behaviors (e.g., emotional expression) to communicate and interact with their children (Zhong et al., 2023). Chinese familial structures distinguished by extended periods of parent-child cohabitation and culturally specific parenting practices—such as the emphasis on collectivist values and academic achievement. In Chinese families, children typically live the longest and have the closest relationship with their parents. Parents are children's first teachers, and their parental rearing style affects children's personality formation and social interpersonal relationships (Basso et al., 2019; Zhang et al., 2022). Furthermore, China's one-child policy and the disparities between urban and rural areas introduce demographic variables that may differentially influence the development of students' social adaptability. Additionally, the evolving social dynamics, characterized by the increasing prominence of digital platforms and virtual interactions, play a pivotal role in shaping interpersonal skills. With the advancement of internet technology, various online games and social media have become prevalent among students, which inevitably affects their psychological, social, and school functions and maybe damage their social adaptability development (Chou et al., 2017; Van Den Eijnden et al., 2018). Therefore, understanding the current status and related influencing factors of medical students' social adaptability is necessary.

Previous studies have documented the correlation between parenting rearing styles and children's competence outcomes. Specifically, parental warmth has been found to positively influence children's psychological adjustment, whereas parenting rearing styles characterized by strictness, control, and indulgence tend to yield the opposite effect (Chen et al., 2000; Garcia et al., 2020; Gniewosz et al., 2023). Additionally, socioeconomic factors, including parental occupations, educational levels, and household income, have also been linked to children's psychological adjustment (Abdul Karim et al., 2022). Psychological factors, such as social anxiety, have been negatively associated with adolescents' psychological adjustment, subsequently impacting their social adaptability (Song and Ding, 2024). Despite these findings, there is a paucity of research specifically examining the impact of parental rearing styles on the social adaptability of medical college students, particularly regarding the mediating role of social psychology process in this context. Based on this premise, we hypothesize that parental rearing styles may influence the social adaptability of medical students by affecting their social psychological processes.

This study aims to evaluate current status of parental rearing styles, social psychology process, and social adaptability among Chinese medical students. It seeks to identify key factors, particularly in parenting styles and social psychology process, that impact students' social adaptability, and further explored how social psychology process mediates the relationship between parental rearing styles and social adaptability. Additionally, the collective influence of familial and socioeconomic factors on the social adaptability of Chinese medical students also examined. The findings are significant for enhancing the competency of medical students as qualified healthcare professionals. Furthermore, our results provide evidence-based strategies to refine medical education policies, highlighting the importance of familial and social psychology process interventions alongside curricular reforms in medical colleges. This approach also actively responds to urgent calls for holistic competency development in global medical education.

## Method

A cross-sectional survey study was conducted, and the questionnaire was designed and distributed to medical college students via the online questionnaire investigation program “Questionnaire Star”. The participants were from medical colleges or universities such as Jilin Medicine University, Beihua University, and Baicheng Medical College, all in Jilin Province. The investigation was performed from January 2023 to April 2023, and a total of 2,215 college students participated in this survey. Of these, 2,066 students majoring in medical-related fields were included in the analysis, while 149 students from non-medical-related disciplines were excluded.

The contents of the questionnaire comprised information about basic demographic characteristics, the parental rearing style, social adaptability, social psychology process (including social avoidance and distress), and personality characteristics of the recruiters. The status of the personality characteristics of the participants was not included in present analysis. The revised Chinese version of parental rearing style survey scale (EMBU, Egma Minnen av Bardndos nauppforstran) co-complied by Perris et al. (1980) was used to evaluate the parental rearing pattern of the participants (Perris et al., 1980; Yue et al., 1993). The questionnaire contained 66 questions and considered the dimensions of rearing styles of fathers and mothers. The father dimension comprised six factors; factor I: emotional warmth and understanding, included questions such as, “I can feel my parents‘ love for me”; factor II: punishment and strictness, included questions such as, “My parents punished me even for small mistakes”; factor III: excessive interference, included questions such as, “I feel my parents interfere in everything that I do”; factor IV: preference, included questions such as, “My parents dote on me more than my brothers and sister”; factor V: rejection and denial, included questions such as, “Parents are very stingy when it comes to meeting my needs”; factor VI: overprotection, included questions such as, “My parents always worry too much about my health”. The mother dimension of rearing style comprised five factors: factor I: emotional warmth and understanding, included questions such as, “I think my parents respect my point of view”; factor II: excessive interference and overprotection, included questions such as, “My parents always dictate what I should wear or how I should dress”; factor III: rejection and denial, included questions such as, “My parents always think that I am the cause of their unhappiness”; factor IV: punishment and strictness, included questions such as, “When I was a child, my parents often beat me or scold me in front of others”; factor V: preference, included questions such as, “My parents can give me some things that my other brothers or sisters can't”. The responses to each question were categorized into four levels: “Never,” “Occasionally,” “Often,” and “Always,” with scores assigned from 1 to 4. Participants who experienced the absence of one or both parents during their childhood were instructed to complete the corresponding column for either the father or the mother. Additionally, respondents who are only children and have no siblings were exempted from answering the related questions. The coefficients of Cronbach α of the overall EMBU scale was 0.920. The coefficients of Cronbach α in dimensions of father's factors I–VI were 0.922, 0.934, 0.694, 0.758, 0.871, and 0.704, respectively; for dimensions of mother's factors I–V, the values were 0.941, 0.839, 0.912, 0.930, and 0.758, respectively.

The evaluation of the social adaptability of the participants adopted the Social Adaptability Diagnostic scale (SAD) that was compiled by Professor Zheng Richang of Beijing Normal University (Zheng, 1999). The scale comprised 20 questions, included questions such as, “Every time I go to a new place, it is easy for me to get close to others”, “The more crowded the place, the more nervous I get”. The answers were “yes” “not sure” and “no” corresponding to the scores of −2, 0, and 2 in the odd-numbered questions or 2, 0, and −2 in the even-numbered questions, respectively. A high SAD score indicates strong social adaptability. The coefficients of Cronbach α of the overall SAD scale was 0.797.

The social avoidance and distress scale (SADS) compiled by Watson and Friend (1969) was used to measure the social avoidance and distress feelings of individuals in their daily lives (Watson and Friend, 1969). Social avoidance and distress refer to individuals' persistent and significant avoidance tendency toward social interactions and negative emotions and distress in a social environment (Zhao et al., 2022). The SADS comprises 28 questions, of which 14 assess social avoidance and the remaining 14 assess participants' social distress. For example, for the dimension of social avoidance, included questions such as, “I try to avoid situations which force me to be very sociables”, “If the chance comes to meet new people, I often take it”. For the dimension of social distress, included questions such as, “I often find social occasions upsetting”, “I usually feel relaxed when I am with a group of people”. Each question was scored from 1 to 0. The participants answered “yes” or “no” based on their own actual situation. “Yes” was scored 1 point, and “no” was scored 0 points. However, for questions 1, 3, 4, 6, 7, 9, 12, 15, 17, 19, 22, 25, 27, and 28, these fourteen items were scored in reverse, that was, “yes” was scored 0 points and “no” was scored 1 point. Add up the scores of the 28 items to get the total score. Scores ranged from 0 (the lowest level of avoidance and distress) to 28 (the highest level of avoidance and distress). The coefficients of Cronbach α of the overall SADS scale was 0.879, while the coefficients of Cronbach α in social avoidance and distress dimensions were 0.768 and 0.801, respectively.

The well-established questionnaire in “Questionnaire Star” was disseminated to medical-related college students via the class monitors or study committee members, utilizing internet application such as WeChat or QQ groups. The contents of the questionnaire did not involve private information about the respondents, and the investigation was performed using a voluntary and anonymous approach. Before the questionnaires were distributed, the purpose of the investigation was explained to potential respondents, whom were informed that the investigation was based on the principles of confidentiality, respect, and voluntary participation. The respondents voluntarily participated in the survey and completed the questionnaires based on their circumstances. In accordance with these principles, participants were not required to sign an informed consent document. Completion of the questionnaires was considered as provision of their informed consent.

The data were analyzed using R(v4.3.1), SPSS(v28.0), and Amos(v23.0) (SPSS Inc., Chicago, IL). The quantitative variables are presented as means (Mean) and standard deviations (SD), and the categorical variables are presented as numbers (N) and percentages (%). Analysis of variance, the non-parametric Wilcoxon rank sum test, or the Kruskal-Wallis rank sum test method was performed to reveal the difference in scores of each scale among demographic characteristic variables. Multiple linear regression was used to analyze the potential factors influencing the social adaptability of medical college students, with elimination and entry standards of 0.05 and 0.10, respectively. The standardized regression coefficient was also calculated to contrast the effect of the screened independent variables. The difference was statistically significant if the *P value* was less than 0.05. Prior to conducting the analysis, the model's assumptions, including linearity, homoscedasticity, independence of errors and normality of residuals were thoroughly verified and confirmed to be satisfied. Mediation effect models were also developed to further explore the mediating role of social psychology process in the relationship between parental rearing styles and social adaptability among medical college students. The standardized coefficients (β values) were calculated to assess the magnitude of the influence exerted by parental rearing styles and social psychology process on social adaptability.

## Results

### Basic demographic characteristics of medical college students

Among the 2,066 participants, the mean and standard deviation of age was 20.38 ± 1.82 years; 1,415 were female, accounting for 68.5%; 1,336 were rural residents, accounting for 64.7%; 1,237 were from one-child families, accounting for 59.9%; the primary educational level of their parents was junior high school or above, accounting for 40.4% and 38.4%, respectively (Table 1).

**Table 1:** Basic demographic characteristics of the recruited medical college students.

**Characteristic**	**Mean (*SD*); *n* (%)**
**Age(years)**	20.38 (1.82)
**Gender**	
Male	651 (31.5)
Female	1415 (68.5)
**Census register**	
Urban	730 (35.3)
Rural	1336 (64.7)
**Only child**	
Yes	829 (40.1)
No	1237 (59.9)
**Father's education level**	
College or above	331 (16.0)
Technical secondary or senior high school	542 (26.2)
Junior high school	834 (40.4)
Primary school	359 (17.4)
**Mother's education level**	
College or above	285 (13.8)
Technical secondary or senior high school	493 (23.9)
Junior high school	794 (38.4)
Primary school	494 (23.9)
**Total**	2,066 (100.0)

### Status of parental rearing style of medical college students

The difference in scores of the six factors related to the rearing style of fathers among the demographic characteristic variables was analyzed. The results show that students identified as female, urban households and one-child families exhibited high scores for factor I (emotional warmth and understanding) (*p* < 0.05). In addition, the higher the educational levels of the parents, the higher the scores of students in terms of factor I (*p* < 0.001). Regarding factors II (punishment and harshness), III (excessive interference), V (rejection and denial), and VI (overprotection), male students exhibited higher scores than female students (*p* < 0.001). These results indicate a high likelihood of male students experiencing severe and rigorous rearing styles from their fathers. Students from urban households and one-child families and those whose parents had high educational levels exhibited high scores for factor IV (preference; Table 2).

**Table 2:** Scores of paternal parenting style among different demographic characteristics in medical college students.

**Characteristic**	**Paternal rearing style dimensions**																	
	**Factor I**			**Facto II** ^*^			**Factor III**			**Factor IV**			**Factor V** ^*^			**Factor VI**		
	**Mean**	**SD**	***p-value***	**Mean**	***SD***	***p-value***	**Mean**	***SD***	***p-value***	**Mean**	***SD***	***p-value***	**Mean**	***SD***	***p-value***	**Mean**	***SD***	***p-value***
Gender																		
Male	51.90	11.59	0.008	20.57	7.20	<0.001	21.08	4.56	<0.001	10.72	3.54	0.206	10.66	3.77	<0.001	10.7	2.88	<0.001
Female	53.32	11.28		17.86	6.12		19.58	4.62		10.52	3.36		9.51	3.36		9.84	2.71	
Census register																		
Urban	55.02	12.03	<0.001	18.69	6.79	0.414	20.21	4.97	0.266	11.37	3.81	<0.001	9.90	3.63	0.851	10.2	2.95	0.297
Rural	51.70	10.85		18.72	6.50		19.97	4.47		10.15	3.10		9.86	3.48		10.07	2.70	
Only child																		
Yes	54.76	12.24	<0.001	18.79	6.74	0.994	20.24	5.09	0.135	11.89	3.93	<0.001	9.95	3.75	0.777	10.21	3.01	0.216
No	51.61	10.61		18.66	6.51		19.93	4.33		9.71	2.69		9.82	3.39		10.05	2.64	
Father education																		
College or above	56.31	12.15	<0.001	18.26	6.86	0.059	20.27	5.20	0.585	11.40	3.97	<0.001	9.75	3.67	0.206	10.24	3.16	0.796
Technical secondary or senior high school	53.14	11.60		18.97	6.95		20.30	4.94		10.82	3.48		10.01	3.74		10.15	2.86	
Junior high school	52.48	10.94		18.52	6.34		19.62	4.22		10.33	3.23		9.74	3.36		9.95	2.66	
Primary school	50.20	10.60		19.16	6.40		20.51	4.57		10.05	3.01		10.11	3.48		10.32	2.64	
Mother education																		
College or above	56.27	12.22	<0.001	18.74	7.31	0.479	20.34	5.51	0.107	11.29	4.08	<0.001	9.91	3.78	0.564	10.34	3.14	0.413
Technical secondary or senior high school	53.98	11.85		18.85	6.92		20.37	5.05		11.08	3.57		9.92	3.76		10.14	3.07	
Junior high school	52.47	10.99		18.50	6.26		19.78	4.17		10.47	3.29		9.76	3.39		9.97	2.57	
Primary school	50.44	10.44		18.89	6.37		20.02	4.43		9.86	2.84		9.99	3.39		10.19	2.63	

^*^Wilcoxon rank sum test or Kruskal-Wallis rank sum test.

The difference in scores of the five factors related to the rearing style of mothers among demographic characteristic variables was also analyzed. The results show that female students and those from urban households and one-child families exhibited high scores for factor I (emotional warmth and understanding, *p* < 0.05), which also increased with the improvement of their parents' educational levels (*p* < 0.001). Regarding factors II (excessive interference and overprotection), III (rejection and denial), and IV (punishment and severity), male students exhibited higher scores than female students (*p* < 0.001). In accordance with the rearing style of their fathers, the male students also likely experienced severe rearing patterns from their mothers. In addition, students from urban households and one-child families and those whose parents had high educational levels exhibited higher scores for factor V (preferences) than those from rural households and non-one-child families and whose parents had low educational levels (*p* < 0.001; Table 3).

**Table 3:** Scores of maternal parenting style among different demographic characteristics in medical college students.

**Characteristic**	**Maternal rearing style dimensions**														
	**Factor I**			**Factor II**			**Factor III** ^*^			**Factor IV** ^*^			**Factor V**		
	**Mean**	***SD***	***p-value***	**Mean**	***SD***	***p-value***	**Mean**	***SD***	***p-value***	**Mean**	***SD***	***p-value***	**Mean**	***SD***	***p-value***
Gender															
Male	52.02	12.23	0.010	33.89	7.50	<0.001	14.08	5.13	<0.001	15.02	5.70	<0.001	10.72	3.54	0.206
Female	53.46	11.47		31.35	7.52		12.38	4.52		12.83	4.69		10.52	3.36	
Census register															
Urban	55.14	12.3	<0.001	32.48	8.14	0.141	12.86	4.98	0.687	13.50	5.24	0.348	11.37	3.81	<0.001
Rural	51.85	11.24		31.97	7.29		12.95	4.68		13.53	5.07		10.15	3.10	
Only child															
Yes	54.87	12.54	<0.001	32.48	8.34	0.112	12.98	4.97	0.610	13.64	5.28	0.642	11.89	3.93	<0.001
No	51.76	10.99		31.93	7.07		12.87	4.66		13.44	5.03		9.71	2.69	
Father education															
College or above	56.56	12.54	<0.001	32.67	8.50	0.031	12.73	4.97	0.144	13.15	5.29	0.062	11.40	3.97	<0.001
Technical secondary or senior high school	53.28	11.93		32.45	8.01		13.02	5.07		13.70	5.35		10.82	3.48	
Junior high school	52.57	11.25		31.47	6.98		12.77	4.59		13.42	4.98		10.33	3.23	
Primary school	50.36	10.95		32.81	7.41		13.26	4.61		13.82	4.99		10.05	3.01	
Mother education															
College or above	56.48	12.42	<0.001	32.69	8.91	0.462	12.94	5.13	0.366	13.53	5.61	0.407	11.29	4.08	<0.001
Technical secondary or senior high school	54.05	12.31		32.49	8.08		12.99	5.15		13.63	5.34		11.08	3.57	
Junior high school	52.59	11.34		31.72	7.00		12.74	4.55		13.36	4.92		10.47	3.29	
Primary school	50.63	10.73		32.20	7.21		13.12	4.57		13.66	4.97		9.86	2.84	

^*^Wilcoxon rank sum test or Kruskal-Wallis rank sum test.

### Status of social adaptability and social psychology process

The results revealed no significant differences in social adaptability scores among medical students between genders. However, students from urban households and one-child families exhibited higher scores than those from rural households and non-one-child families (*p* < 0.05). The social adaptability scores increased as parents' educational levels increased (*p* < 0.001, Table 4).

**Table 4:** Scores of social adaptability and social psychology process among different demographic characteristics in medical college students.

**Characteristic**	**Social adaptability** ^*^			**Social avoidance** ^*^			**Social distress** ^*^		
	**Mean**	***SD***	***p-value***	**Mean**	***SD***	***p-value***	**Mean**	***SD***	***p-value***
**Gender**									
Male	3.50	11.35	0.077	6.59	3.19	0.052	6.91	3.41	0.012
Female	2.49	12.33		6.91	3.58		7.34	3.75	
**Census register**									
Urban	3.56	13.08	0.038	6.75	3.58	0.567	7.05	3.76	0.149
Rural	2.41	11.41		6.84	3.40		7.29	3.59	
**Only child**									
Yes	3.53	12.82	0.026	6.71	3.51	0.269	6.98	3.80	0.019
No	2.33	11.47		6.88	3.43		7.36	3.54	
**Father education**									
College or above	4.91	13.45	<0.001	6.49	3.80	0.014	6.74	3.94	<0.001
Technical secondary or senior high school	3.17	12.51		6.74	3.46		7.09	3.59	
Junior high school	2.41	11.57		6.85	3.42		7.25	3.64	
Primary school	1.28	10.69		7.13	3.24		7.71	3.45	
**Mother education**									
College or above	5.07	13.05	<0.001	6.35	3.63	<0.001	6.60	3.84	<0.001
Technical secondary or senior high school	3.16	12.79		6.67	3.55		6.89	3.69	
Junior high school	2.80	11.64		6.84	3.37		7.33	3.51	
Primary school	1.18	11.06		7.17	3.41		7.67	3.67	

^*^Analysis of variance.

The social distress scores of female students and those from non-one-child families were higher than those of males and those from one-child families (*p* < 0.05). The social distress and avoidance scores of the students were related to their parents' educational levels. High social distress and avoidance scores were observed for medical college students whose parents had low educational levels (*p* < 0.001) (Table 4).

### Influential factors of medical college students' social adaptability

The independent variables, such as gender, census register, one-child, educational level of parents, social distress and avoidance, and parental rearing style were adjusted. The results of multiple linear regression analysis revealed that social distress (standardized regression coefficient β = −0.399, *p* < 0.001) and social avoidance (β = −0.304, *p* < 0.001) are the most influential negative factors affecting medical college students' social adaptability. The emotional warmth and understanding imparted by their mothers had a significant positive impact on the social adaptability of the students (β = 0.135, *p* < 0.001). Overprotection from their fathers had a significant negative impact on the social adaptability of medical college students (β = −0.087, *p* < 0.001; Table 5).

**Table 5:** Multiple linear regression analysis results of social adaptability in medical college students.

**Variables**	β-unstandardized^*^	**SD^#^**	β-standardized^*^	**T-value**	**P-value**
Constant	15.971	1.196		13.357	< 0.001
Social distress	−1.316	0.079	−0.399	−16.696	< 0.001
Social voidance	−1.056	0.084	−0.304	−12.623	< 0.001
Mother's emotional warmth and understanding	0.138	0.016	0.135	8.678	< 0.001
Father's overprotection	−0.376	0.067	−0.087	−5.655	< 0.001

^*^Unstandardized and standardized regression coefficient. ^#^Standard error.

**Figure 1:**
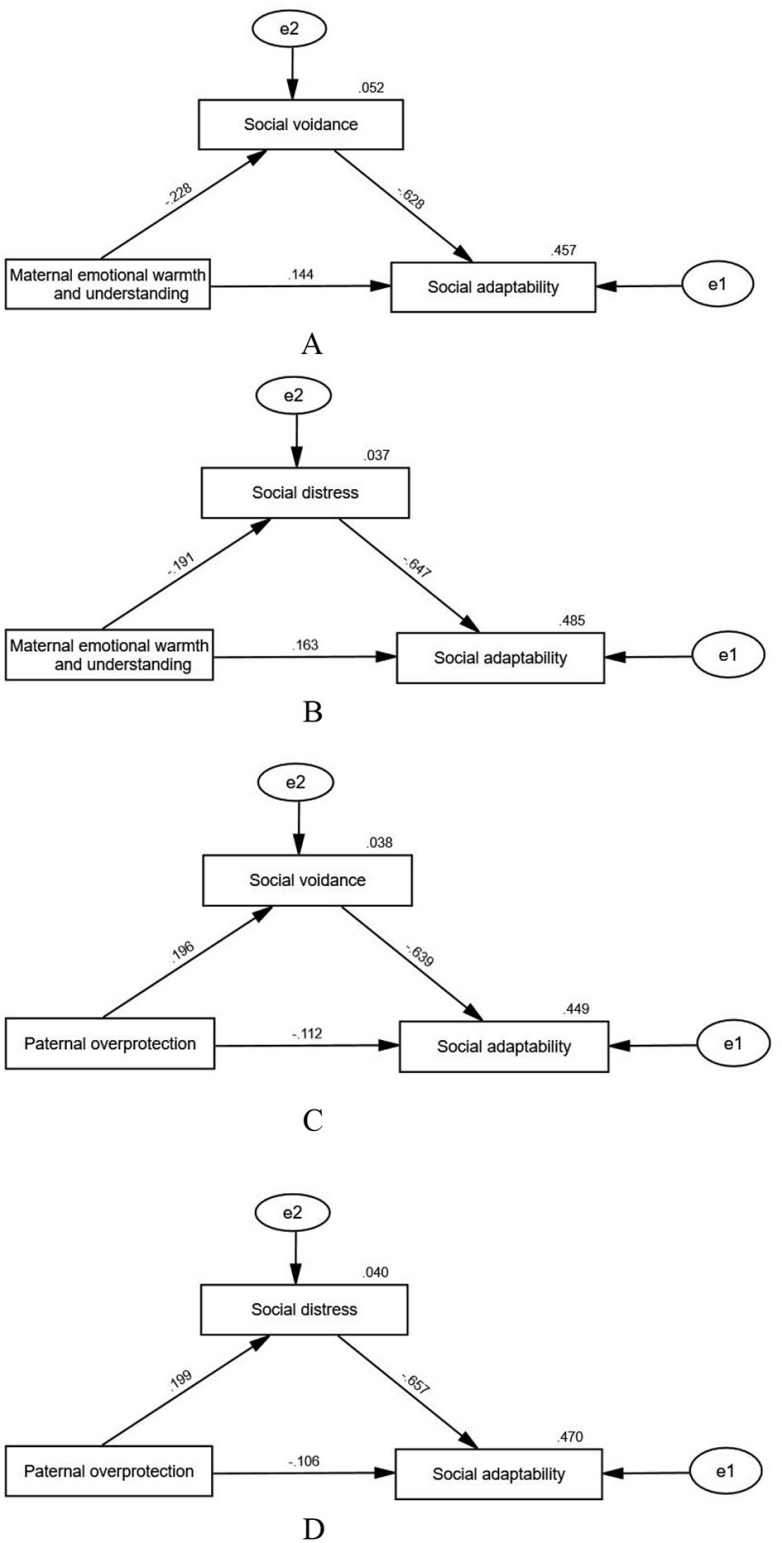
**Four** mediation effect models were established to further explore how social psychology process mediates the relationship between parental rearing styles and social adaptability in medical college students. The fit indices of model **(A-D)** in CFI vaule all was 1, while the RMSEA vaule was 0.470, 0.483,0.460 and 0.474 respectively. The models were just-identified (saturated) with zero degrees of freedom, resulting in perfect fit indices (CFI=1) and undefined RMSEA values; the reported RMSEA values should be interpreted with caution in this context.

Mediation effect models were established further to explore the relationship among parental rearing styles, social psychology process, and social adaptability among medical college students. The results showed a negative correlation between emotional warmth and understanding of rearing style from the mother dimension and social avoidance (β = −0.228, *p* < 0.001) and distress (β = −0.191, *p* < 0.001), whereas overprotection from fathers' rearing style was positively correlated with social avoidance (β = 0.196, *p* < 0.001) and distress (β = 0.199, *p* < 0.001). The parental rearing style can directly or indirectly affect the social adaptability of students through social psychology process; however, its impact on social psychology process is greater. Overall, social avoidance and distress exhibited a greater direct effect than parental rearing style on medical students' social adaptability (see Figure 1).

## Discussion

Several studies examining cross-ethnic and geographically diverse populations have demonstrated a significant correlation between parenting styles and specific achievements in their children (Aunola et al., 2000; Pinquart and Kauser, 2018). However, there is a paucity of research investigating the relationship between parenting styles and adolescents' social adaptability, particularly within the group of medical students. Chinese students primarily study at school from their childhood; notably, medical college students typically require more time than other students in schools due to the long medical education system (Wang, 2021). The competency of social adaptability is particularly important for medical students, and it not only directly affects the quality of medical service but is also related to the emergence of doctor—patient relationships (Xiu et al., 2016). However, current Chinese higher medical education generally lacks targeted courses that cultivate social adaptability for medical college students. The social adaptability of individuals depends on their life experiences and can be continuously enhanced through the accumulation of personal experiences. Family and school are two important growth environments for adolescents to develop social adaptation (Yin et al., 2021); therefore, education from family and school is necessary to strengthen medical college students' social adaptability.

Our findings revealed no significant differences in social adaptability between male and female medical students. This lack of disparity may be attributed to the evolving societal norms regarding gender roles in China, especially among highly educated individuals in urban areas, who constituted the entire of our sample. As expectations for professional competence and interpersonal effectiveness become increasingly gender-neutral, the measures of social adaptability may correspondingly reflect this shift. The social adaptability of medical students from urban households and one-child families was better than that of those from rural households and non-one-child families. This phenomenon may indicate more profound socioeconomic disparities. Urban families frequently have access to greater economic resources and fewer caregiving burdens, enabling them to provide more supportive environments for their children to the additional opportunities of contacting and participating in more social activities and learn more extracurricular cultural knowledge than the rural students, thereby contributing to the promotion and enhancement of their social adaptability. One-child status with higher social adaptability may reflect the “resource concentration effect”. One-child families were mostly urban households (accounting for 59.6%), and parents from one-child families could afford the additional experience and economic expenditure to promote their children's social adaptability competence.

Some studies have demonstrated that social avoidance is currently a clinically worrisome phenomenon for adolescents (Simon et al., 2021; Clifford et al., 2022; Zhu et al., 2023). Although no significant difference in social avoidance performance was observed between male and female medical students in the present study, female students exhibited more serious social distress than male students. In addition, the social distress scores of medical students from non-one-child families were significantly higher than those of students from one-child families. These results indicate that increased attention to social psychology process for female and non-one-child medical college students should be provided.

In addition, as their parents' educational levels increased, the social adaptability of medical students increased. Parental education status is an important influential factor in the self-growth of children (Aelenei et al., 2023). Higher levels of parental education are usually associated with increased exposure to contemporary individualistic values, which may promote more egalitarian and emotionally responsive parenting practices, such as more incline to exemplify effective communication strategies, promote critical thinking, and encourage proactive problem solving skills, which are directly transferable to enhance their children's social adaptability. Furthermore, parents with high educational levels typically have superior economic conditions and are willing to pay extra to cultivate their children's comprehensive adaptability. According to the results, the social avoidance and distress scores of medical students exhibited a decreasing trend with the improvement in their parents' educational levels. Educational level is typically related to an individual's cognition, socioeconomic status, and children's educational concept. Parental socioeconomic status is highly influential in determining a child's physical and mental health and future outcomes (Vukojevic et al., 2017). Therefore, the relationship between the educational status of parents, the social adaptability of their children, and the occurrence of social and psychological problems among medical students is reasonable.

Considering parental rearing style, male medical students exhibited higher scores than female medical students for the factors of “punishment and severity,” “excessive interference,” and “rejection and denial.” This result suggests that parents are more inclined to adopt strict family rearing styles for their sons than for their daughters. Students from urban households and one-child families exhibited higher “emotional warmth and understanding” and “preference” scores than those from rural households and non-one-child families. This finding shows an increasing tendency with parental educational levels, indicating that parents with high educational levels and from urban households or one-child families prefer a mild rearing style for their children.

The results of the multivariate analysis and mediation effect model indicate that social avoidance and distress are independent and the strongest negative factors influencing the social adaptability of medical college students. The mediating effect model results confirmed parental rearing style can directly or indirectly affect students' social adaptability through social psychology process such as social avoidance and distress, which elucidates a cascading pathway, wherein parenting rearing styles influence psychological states, and subsequently determine students' social adaptability outcomes. A cross-cultural study has demonstrated a substantial linkage between parental rearing style and adolescent mental health (Weitkamp and Seiffge-Krenke, 2019). Thus, understanding students' social psychological status and providing targeted psychological consultations or intervening services are ways for medical college teachers to prevent students from suffering social psychological problems and subsequently benefit for developing social adaptability.

The “understanding and emotional warmth” rearing style from the mother dimension is an independent factor influencing medical students' social adaptability. Traditionally, mothers are linked with nurturing roles and providing emotional support. Parental emotional warmth seems to mitigate stress by enhancing their children's emotional regulation, thereby fostering confidence in novel social interactions and enabling them to approach social challenges with resilience. Study had showed the emotional warmth of parental rearing style can effectively reduce the occurrence of neglect, which can affect the physical and mental health of adolescents (Wang et al., 2016). The mediation effect model revealed that the rearing style of “understanding and emotional warmth” of mothers may exert a positive effect on solving offspring's psychological problems. When students encounter setbacks or failures in their daily lives, understanding and emotional warmth from mothers will facilitate confidence building to overcome difficulties and challenges, thereby improving the social adaptability competence of the children.

The rearing style of “overprotection” from fathers was found to be a negative factor influencing the social adaptability of medical students. This phenomenon is particularly noteworthy given China's traditional collectivist cultural norms, which prioritize parental authority and often emphasize conformity over individual agency. Within Chinese families, fathers frequently assume the role of moral and behavioral disciplinarians, focusing on control and supervision rather than emotional expression in child-rearing practices. While this parenting style can effectively shield children from potentially hazardous situations and is aimed at ensuring conformity and success. It simultaneously restricts the development of self-efficacy and social initiative, which are crucial for fostering social adaptability. It also limits opportunities for children to cultivate autonomy and engage in independent problem-solving and experiential learning, the essential components of social adaptability. In contrast, maternal understanding and emotional warmth may serve as a mitigating factor against the stringent expectations imposed by fathers, helping to alleviate some of the negative consequences associated with an overly controlled upbringing. Consequently, this form of “overprotection” in parenting is likely to hinder the development of children's social adaptability.

Although this study is a cross-sectional study, the parental rearing style is typically perceived as a type of long-term and stable pattern. Parental rearing styles and their consistency are related to emotional and behavioral status in early adulthood (Baker and Hoerger, 2012; Zou et al., 2020). Consequently, the observed associations between parental rearing styles and social adaptability among medical college students may be regarded as a causal association in certain degree. Even though, we also strongly advocate for future research to utilize longitudinal designs to monitor the progression of social adaptability over time and to elucidate the temporal dynamics among parenting styles, social psychology process, and social adaptability outcomes. Furthermore, interventional studies are necessary to evaluate the effectiveness of programs designed to modify parental rearing practices and to mitigate social avoidance and distress for promoting students' social adaptability.

Through this large-sample investigation, present study identified numerous important factors influencing medical college students' social adaptability competence. However, this study has some shortcomings. First, the survey was limited to students from several regional universities or colleges in Jilin Province, China, which may limit the generalizability of relevant results. Second, the Cronbach's α value for the father's rearing style subscale in excessive interference was 0.694, which is marginally below the conventional threshold of 0.7 may lead to the speculation of introducing potential reliability concerns regarding the findings related to gender differences for this subscale. Third, the survey was based on a web-based survey platform program; thus, there may be a self-selection bias, resulting in the participants not being representative, and the results may not reflect the status of all Chinese medical college students. Surveys that involve multi-geographic or more representative samples should be conducted in future studies to further validate the findings of the present study.

The contribution of this study is its integration of familial, psychological, and sociodemographic factors within the context of the evolving landscape of medical education in China. It revealed that strengthening parental rearing styles of emotional warmth and understanding, avoiding social psychological problems such as social avoidance and distress, and preventing overprotective parental rearing styles can improve medical college students' social adaptability. From a practical standpoint, these findings necessitate a collaborative approach involving both families and educational institutions. It is recommended that parenting workshops be established to inform parents about the negative consequences of overprotectiveness and the advantages of fostering emotional warmth. Concurrently, it is imperative for medical colleges to integrate psychological support mechanisms and social skills training into their curricula to proactively mitigate issues related to social avoidance and distress.

In conclusion, our study offers actionable insights for family-centered interventions and advances the mechanistic understanding of social adaptability development for Chinese medical students. This theoretical refinement not only elucidates the significance of specific rearing styles but also guides the development of interventions targeting modifiable mediators such as social psychological factors, to enhance social adaptability. These targeted measures should be adopted to improve the social adaptability of Chinese medical college students, these also provide considerable reference value for the medical education globally.

## Data Availability

The raw data supporting the conclusions of this article will be made available by the authors, without undue reservation.
